# The Periodoscope

**Published:** 1848-10-01

**Authors:** 


*The Periodoscope;* with its Application to Obstetric Calculations, and the Periodicities of the Sex. By W. Tyler Smith, M.B. Lond., Obstetric Lecturer in the Hunterian School of Medicine. London. Churchill, Princes-street, Soho.

We received our copy of this work at too late a period to notice it in that portion of our Journal appropriated to miscellaneous reviews; but we cannot go to press without directing the attention of the profession to this important and valuable contribution to obstetric literature. The object of the essay is to elucidate the "*periodicities of the female sex,*" and the periodoscope is constructed with the view of enabling the practitioner to calculate, with almost unerring accuracy, these epochs in the female economy. The obstetric calendar is most ingeniously arranged, and will be found of essential service in elucidating all questions having reference to the duration of pregnancy, and other natural and morbid phenomena peculiar to women. The psychological physician, engaged in the treatment of insanity, can appreciate the value of a guide which will enable him to ascertain, with even an approach to correctness, the normal and disordered condition of the catamenial secretions. How much of the insanity of females is dependent upon or associated with the state of this important function? As the author of this essay observes, "in cases of prolonged puerperal mania, accompanied by suppression of the lochia and catamenia, it is of considerable value to attend to the ovarian periods following parturition. In these distressing cases, when the ovarian nisus comes round without being attended by the uterine secretion, there is generally a furious exacerbation of the malady. The treatment of these periods is often the most important part of the treatment of the whole disease."—(p. 40.) In mania, connected with uterine derangement, it is important for the physician to be able to reckon the respective catamenial periods with accuracy; by the aid of Dr. Tyler Smith's periodoscope this desirable object will be obtained, and it is on this ground principally (for into the other merits of the invention it would be irrelevant for us to enter) that we earnestly recommend those connected with the treatment of the insane to avail themselves of this valuable means of ascertaining the precise state of a function which more or less gives a character to all the diseases peculiar to women.

